# Advancing Metallic Lithium Anodes: A Review of Interface Design, Electrolyte Innovation, and Performance Enhancement Strategies

**DOI:** 10.3390/molecules29153624

**Published:** 2024-07-31

**Authors:** Junwei Shi, Kailin Jiang, Yameng Fan, Lingfei Zhao, Zhenxiang Cheng, Peng Yu, Jian Peng, Min Wan

**Affiliations:** 1 School of Chemical and Environmental Engineering, Wuhan Polytechnic University, Wuhan 430048, China; junweishi0319@163.com (J.S.); jkl6868@163.com (K.J.); 2 Institute for Superconducting and Electronic Materials, Australian Institute for Innovative Materials, University of Wollongong, Innovation Campus, Squires Way, Wollongong, NSW 2522, Australia; yfan@uow.edu.au (Y.F.); lingfeiz@uow.edu.au (L.Z.); cheng@uow.edu.au (Z.C.); 3 State Key Laboratory of Material Processing and Die & Mold Technology, School of Materials Science and Engineering, Huazhong University of Science and Technology, Wuhan 430074, China; 4 Department of Mechanical and Materials Engineering, University of Western Ontario, London, ON N6A 5B9, Canada

**Keywords:** metallic lithium anodes, electrochemical properties, interface design modification, lithium dendrites, high energy density

## Abstract

Lithium (Li) metal is one of the most promising anode materials for next-generation, high-energy, Li-based batteries due to its exceptionally high specific capacity and low reduction potential. Nonetheless, intrinsic challenges such as detrimental interfacial reactions, significant volume expansion, and dendritic growth present considerable obstacles to its practical application. This review comprehensively summarizes various recent strategies for the modification and protection of metallic lithium anodes, offering insight into the latest advancements in electrode enhancement, electrolyte innovation, and interfacial design, as well as theoretical simulations related to the above. One notable trend is the optimization of electrolytes to suppress dendrite formation and enhance the stability of the electrode–electrolyte interface. This has been achieved through the development of new electrolytes with higher ionic conductivity and better compatibility with Li metal. Furthermore, significant progress has been made in the design and synthesis of novel Li metal composite anodes. These composite anodes, incorporating various additives such as polymers, ceramic particles, and carbon nanotubes, exhibit improved cycling stability and safety compared to pure Li metal. Research has used simulation computing, machine learning, and other methods to achieve electrochemical mechanics modeling and multi-field simulation in order to analyze and predict non-uniform lithium deposition processes and control factors. In-depth investigations into the electrochemical reactions, interfacial chemistry, and physical properties of these electrodes have provided valuable insights into their design and optimization. It systematically encapsulates the state-of-the-art developments in anode protection and delineates prospective trajectories for the technology’s industrial evolution. This review aims to provide a detailed overview of the latest strategies for enhancing metallic lithium anodes in lithium-ion batteries, addressing the primary challenges and suggesting future directions for industrial advancement.

## 1. Introduction

In response to global warming and extreme weather events, the worldwide demand for energy continues to grow, while the outlook for traditional energy systems appearing bleak [1,2,3,4]. The development of clean energy, such as low carbon, environmentally friendly, high-performance energy conversion, and storage systems, is essential for enhancing the current energy structure and reducing the use of fossil energy [5,6,7]. As a typical clean energy, lithium batteries offer significant advantages, including high energy density, high discharge power, long cycle life, no memory effect, and environmental sustainability in facilitating the widespread use of portable electronic devices and electric vehicles [8,9]. The unique configuration of lithium pouch cells can lead to differences in pressure distribution, electrode material distribution, and electrolyte distribution, which can affect their failure characteristics [10]. Through innovations in the field of electroplating chemistry, researchers have learned about the deposition and exfoliation behavior of lithium ions and have optimized electrolyte composition and operating conditions, thereby minimizing dendrite growth and improving cycling performance, providing important insights for the study of lithium-ion plating processes in non-aqueous liquid electrolytes to improve the stability of lithium metal anodes [11,12]. Strategies such as the use of composite anodes, the introduction of protective coatings, the optimization of electrolytes, and the integration of lithium metal anodes with advanced cathode materials and separators have shown promising results [13]. Among various advancements, the lithium metal anode is regarded as the most promising candidate for the next-generation of high-energy-density lithium batteries, owning to its mass specific capacity of 3860 mAh g^−1^ and a volume specific capacity of 2065 mAh cm^−3^ [14,15,16]. Additionally, the electrochemical redox potential (−3.040 V vs. standard hydrogen electrode) of the metallic lithium anode is significantly lower than those of other metal anodes [17]. These unique advantages also position metallic Li as one of the most important anode materials in the era of all-solid-state batteries, potentially facilitating a significant leap in the safe and energy density of lithium batteries.

Despite the above-mentioned advantages, with the deepening of the research on lithium metal anode, there are still challenges that need to be overcome before lithium metal anode batteries can be commercialized. Firstly, due to the high diffusion barrier and weak interaction energy, lithium atoms tend to form one-dimensional long tubular or filamentary lithium dendrites [18,19,20]. The constantly growing lithium dendrites can penetrate the separator and reach the cathode, causing internal electrical contact and short circuits in the battery, which may lead to thermal runaway and explosion risks [19,21,22,23]. Secondly, metallic lithium shows thermodynamic instability and is prone to react with solvents or additives in the electrolyte to form a solid electrolyte interphase (SEI) [24]. The typically formed SEIs are brittle and inhomogeneous, resulting in poor anode interface stability [25]. The rupture of the SEI during cycling results in the constant depletion of electrolyte and Li. Furthermore, the electrical insulation of the SEI layer makes it possible for lithium dendrites to detach from the anode and become electrochemically inert, known as “dead lithium”, resulting in low Coulombic efficiency and lithium utilization [26,27]. Lastly, the infinite volume change of the lithium metal anode during deposition/stripping can easily destroy the naturally formed SEI with poor mechanical properties, thus exposing the highly reactive lithium metal to the electrolyte and forming a new interface [28,29]. The continuous destruction and regeneration of SEI not only leads to the ongoing consumption of active lithium and electrolyte but also increases the battery’s impedance, ultimately leading to a rapid decline in the electrochemical performance of the battery [30].

**Figure 1:**
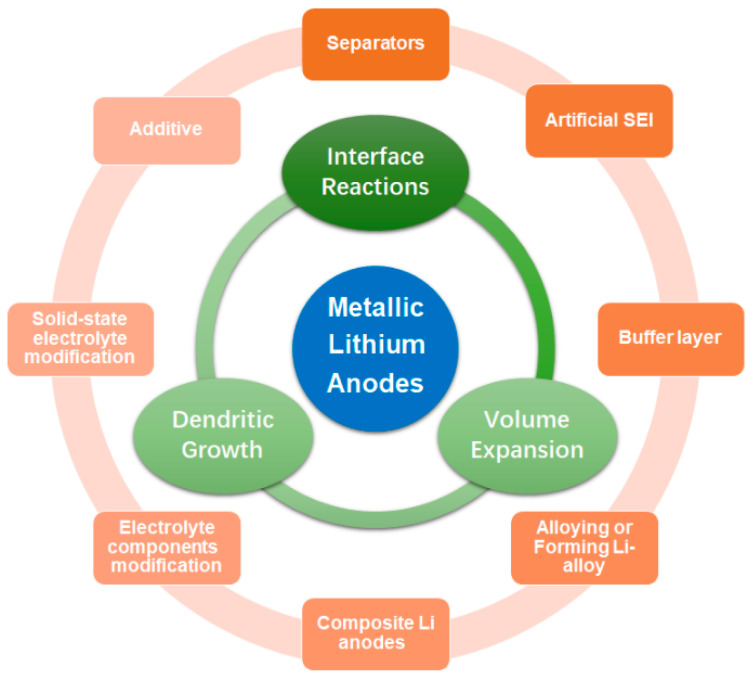
Challenges and improvement strategies for lithium metal anodes.

In summary, studies on metal lithium anodes have identified three major challenges: lithium dendrite growth, uncontrolled interfacial reactions, and significant volume expansion, as shown in Figure 1 [31]. Generally, there are three types of solutions: electrode design, electrolyte modification, and interface engineering. However, with the increasing popularity of research on metal lithium anodes, new discoveries, and modification strategies continue to emerge in recent years. And previous generalizations cannot keep up with the latest progress, especially in novel, convenient, low-cost, and multi new strategies, or provide clear explanations for some issues. A prefatory review article focusing on the modification and protection of anodes can greatly benefit researchers by quickly orienting them towards the current developmental direction of the field [32]. Therefore, this review aims to summarize recent research from a broad perspective, including comprehensive fundamental knowledge and the latest work on metallic Li anodes. Moreover, this work, grounded in a vast array of published studies, offers detailed discussions on specific mechanisms not thoroughly explained in other comments. 

## 2. Strategies for Designing Advanced Li Metal Anode

Beginning from the lithium anode itself, modification strategies and protection measures primarily follows two directions: alloying/forming Li-alloy and designing composite Li anodes. These approaches are essentially effective in guiding nucleation deposition and constraining volume expansion.

### 2.1. Alloying or Forming Li-Alloy

To address issues such as reaction between electrodes and electrolytes, electrode alloying is commonly employed to improve safety, achieve dendrite-free anode, high Coulombic efficiency, and extend cycle lifespan [33]. Significant advancement has been achieved with the development of various Li alloy anodes, including various forms of lithium-carbon alloys (such as graphite-Li hybrid anodes), Li-Al alloys, lithium-zinc alloys, lithium-indium alloys, etc. [34,35,36]. However, challenges related to interface contact, as well as problems arising from stress due to volume expansion and the conduction rate of ions and electrons inside the electrode, have not been adequately addressed.

**Figure 2:**
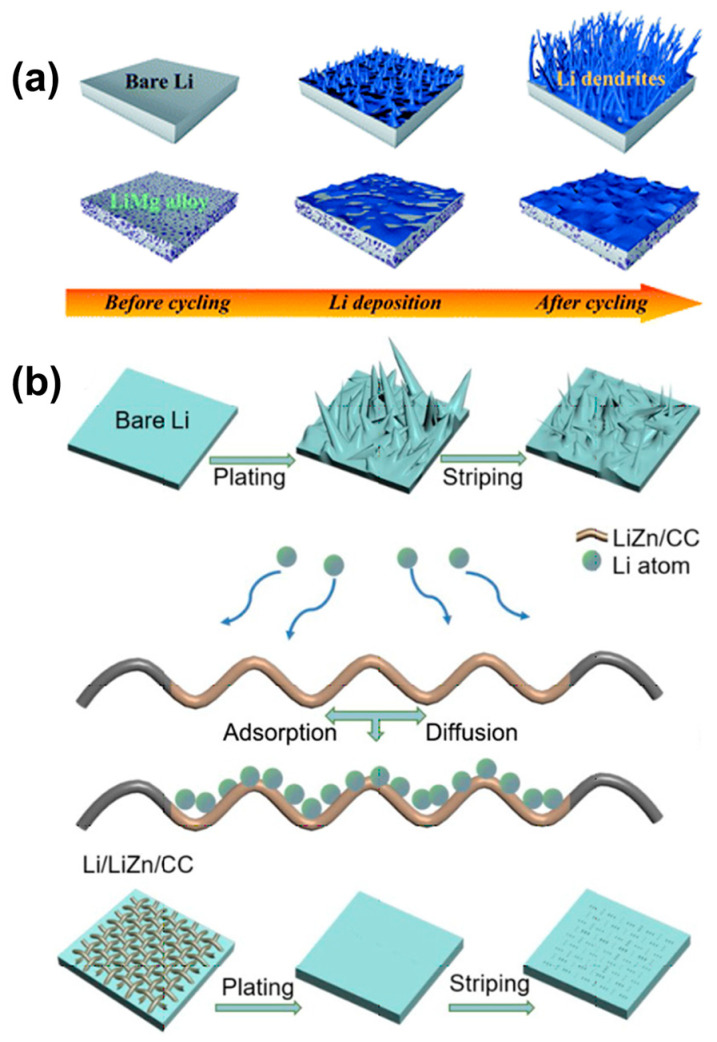
Schematics of (**a**) the inhibition of lithium growth by Li-Mg alloy [39]. (**b**) Li deposition on bare Li or Li/LiZn/CC substrates [40].

The mechanical properties of alloys play a crucial role in controlling dendrite formation on the anode. Compared to lithium-carbon alloys, a slightly higher voltage of lithium-metal alloy anodes contributes to enhancing the interfacial stability with SEs, as shown in Figure 2. Huang demonstrated the importance of the mechanical properties of the alloy in terms of dendrite suppression. Compared to other Li-alloys, the high compressive strength and high Young’s modulus of Li-Al alloy enabled it to effectively maintain the integrity of the electrode during repeated loading and unloading. It was shown that Li-Al alloy anodes exhibited the highest ability to inhibit dendrite formation, and no soft shorts were observed in symmetrical cells under high current densities of 40 mA cm^−2^ and a capacity of 3 mAh cm^−2^, with excellent cycling performance and electrochemical performance. It is expected to be a promising anode for high-rate and high-area capacity solid-state battery (SSBs) [37]. Peng used a one-step phase change alloying reaction to construct a lithium alloy/ionic conductor composite anode. In this process, the original LiMgPO_4_ (LMP) was converted into ionically conductive Li_3_PO_4_ and Mg, and Mg reacts with Li to form Li-Mg alloys to form Li-LMP composites. On the one hand, the alloying of nanoscale Li_3_PO_4_ improved the mechanical strength of Li-LMP, enabling it to withstand the local stresses that would occur during cycling, and on the other hand, it enhanced the ion transfer inside Li-LMP and accelerated the dynamic mass transfer process. Li-LMP exhibited higher rate performance and cycling stability in both ether and carbonate electrolytes [38]. Guo constructed a solid solution-based Li-Mg alloy framework through mechanical rolling, which acted as an electron/ionic double conductive lithium host, inhibiting lithium dendrites and volume changes to stabilize the metal lithium anode. The lithophilic Li-Mg solid solution alloy promoted uniform Li deposition and promoted Li extraction during the peeling process, which inhibited the parasitic reaction and dendrites on the surface of the Li metal anode [39]. Zheng chose 3D carbon cloth (CC) as the substrate to cover the entire zinc oxide quantum dots to inhibit Li dendrite growth. The spontaneous reaction between ZnO QDs and molten Li formed LiZn alloy and covered the CC surface, so the substrate had a high Li adsorption affinity and appropriate Li diffusion barrier energy. The three-dimensional structure of CC provided a higher specific surface area and reduced the effective current density of the electrode, and the surface of the formed LiZn alloy had a high adsorption energy (−1.89 eV) and a diffusion barrier suitable for Li atoms (0.68 eV), which was conducive to the uniform dense nucleation and deposition of Li. Due to the dendrite-free Li deposits, the Li/LiZn/CC anode worked for 2700 h at 1 and 10 mA cm^−2^ for 2700 and 1900 h, respectively, showing remarkable stability in symmetrical cells [40]. 

### 2.2. Composite Li Anodes

**Figure 3:**
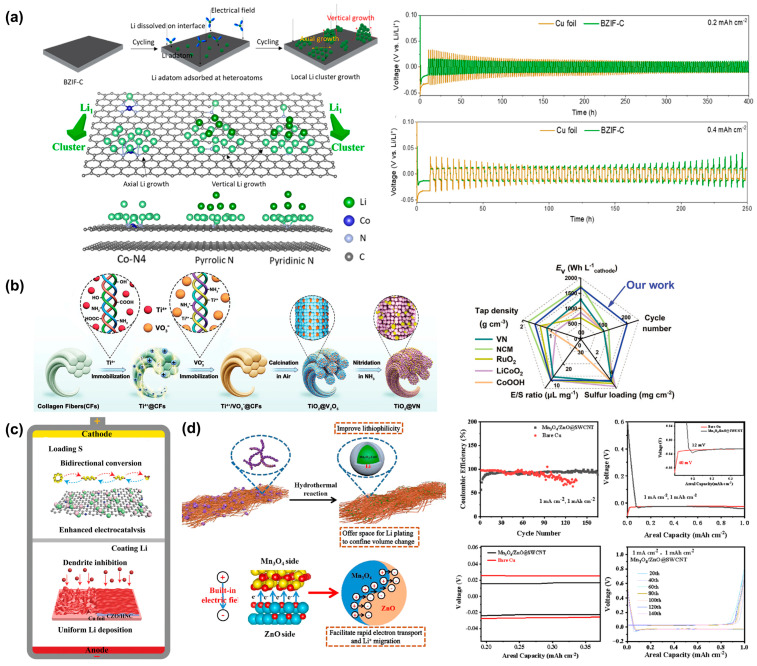
Schematic representation of (**a**) the deposition and growth during the Li plating process with its electrochemical performance [41]. (**b**) The synthesis procedure of fibrous TiO_2_@VN heterostructure with its electrochemical performance [52]. (**c**) The role played by CZO/HNC in the Li-S full cell [53]. (**d**) The synthesis of Mn_3_O_4_/ZnO@SWCNT with its electrochemical\performance [54].

Composite lithium anodes employing 3D host materials with pre-stored lithium properties, or 3D current collectors featuring lithophilic properties, have been demonstrated as effective methods to facilitate the uniform deposition of lithium and prevent the formation of dendrites. This approach can effectively mitigates issues such as irregular lithium deposition, serious volume changes, and safety hazards, as shown in Figure 3 [14]. And the specific data are shown in Table 1.

Qutaish calculated the influence of different heteroatoms (pyridinic N, pyrrolic N, quaternary N, and Co−N_4_) on the growth mechanism of Li clusters using density functional theory: Li grew axially on the surface of the carbon skeleton, while other heteroatoms, such as nitrogen defects, led to the vertical growth of Li [41].

Zhang prepared a 3D Ti_3_C_2_T_x_@Cu current collector where the 3D structure not only effectively reduced the average current density but also alleviated volume expansion. Ti_3_C_2_T_x_ nanosheets as a Li affinity agent facilitated uniform deposition and dendrite-free structure, resulting in a reduction in nucleation overpotential and an increase in Li nucleation sites. After prolonged plating/stripping, the Li-Ti_3_C_2_T_x_@Cu anode’s surface could still maintain uniformity. Under the condition of 1 mA cm^−2^ current density and 1 mAh cm^−2^ surface capacity, the super-stable cycling time of the Li-Ti_3_C_2_T_x_@Cu symmetric cell was about 950 h [42]. Yang electroplated a 3D micro-porous structure of a lithium-friendly nickel scaffold on a flat copper with nano-scale surface roughness to serve as a metal lithium anode collector. The high specific surface area and nanoscale rough surface allowed for low local current density and uniform electric field distribution, which facilitated the uniform deposition of lithium metal at high current densities. The Ni(OH)_2_ thin layer formed in situ during electrodeposition exhibited high lithium affinity, inducing uniform nucleation of lithium. This 3D lithiophilic Ni micro-porous (3D NMV) matrix endowed the Li metal anode with long cycle performance exceeding 830 cycles under an ultra-high current density of 10 mA cm^−2^ [43]. Using low-cost bamboo as a raw material, a nickel-embedded porous graphite carbon fiber (PGCF@Ni) advanced anode was constructed by Wang. Due to its excellent conductivity and high specific surface area, lithium could be uniformly deposited/stripped on the prepared SEI@Li/PGCF@Ni anode without lithium dendrites. In a symmetrical battery, under the conditions of 1 mA cm^−2^/1 mAh cm^−2^, the overpotential after 2000 h was only about 10 mV. The Li-S full battery assembled through collaborative design (SEI@Li/PGCF@Ni||PGCF@Ni/S) exhibited excellent stability, with a capacity retention rate as high as 77.9% after 600 cycles at 1 C [44]. Chen effectively guided uniform Li deposition in the 3D subject by applying magnetron sputtering to uniformly anchor the liphyphilic silver layer on the copper net (Li@/Ag) and realized the spatial control of Li nucleation. The symmetrical battery could maintain a low overpotential (230 mV) and long cycle life (90 h) at a high current of 10 mA cm^2^ with a plating amount of 3 mAh cm^2^. Also, Li@/Ag||LiCoO_2_ cells exhibited high capacity retention (86.39%) after 150 cycles at 2 C [45]. Liu devised a 3D ZIF-8@RGO scaffold material that could function both as a current collector and a host for the metal lithium anode. The preferential deposition of Li was achieved through the synergistic effect of the lithiophilic N/Zn nucleation sites and the independently conductive RGO. The Li/ZIF-8@RGO anode was able to operate for 600 h (pre-loaded with 5 mAh cm^−2^), without short-circuiting, and demonstrated high Coulombic efficiency of up to 98.48% over 350 cycles. The full battery also exhibited excellent cycle stability and rate capability [46].

**Figure 4:**
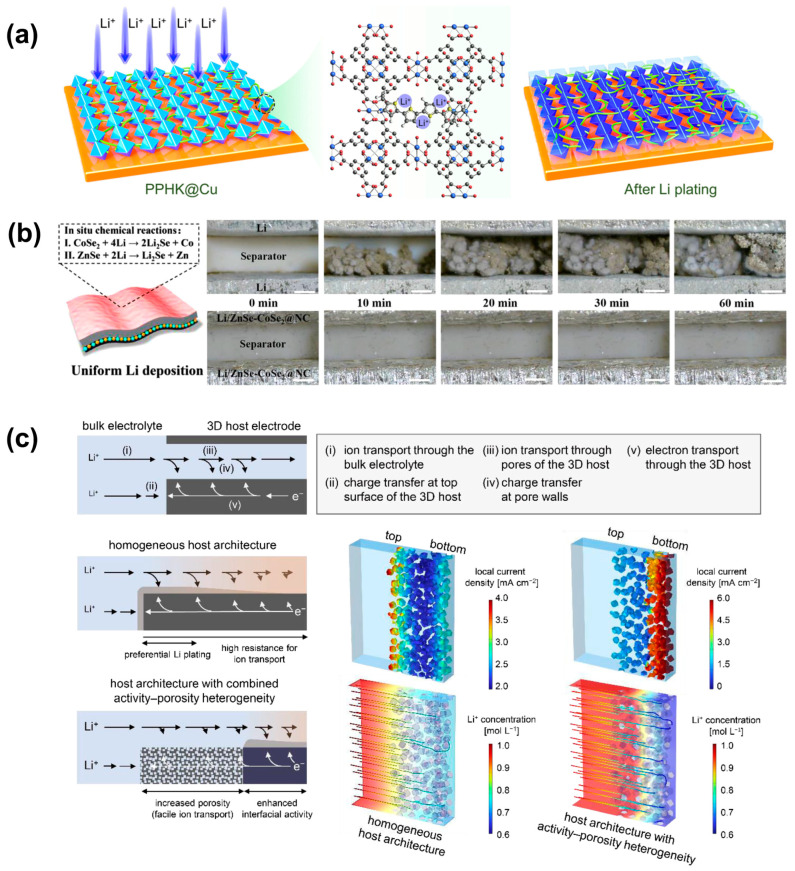
Schematic diagram of (**a**) a strategy to promote homogeneous Li deposition through the “blockchain” synergy via PPy-filled HKUST-1 [47]. (**b**) The structure of two-in-one ZnSe-CoSe_2_@NC host on anode and in situ observations of symmetric cells with pure Li and Li/ZnSe-CoSe_2_@NC electrode cycling at a current density of 3 mA cm^−2^ [49]. (**c**) The multiple reaction steps involved in Li plating in a 3D host architecture [51].

The use of blockchain technology as a tool for process management can enhance the safety of the manufacturing and transporting lithium-ion batteries, as shown in Figure 4. As reported by Ma, who reported the utilization of a conductive polymer-filled metal-organic framework (MOF) as the host for lithium ions, polypyrrole (PPy) acted as a ‘link’ to connect stored lithium ‘blocks’ in the MOF pores. When highly conductive PPy guided fast Li^+^ penetration/extrusion and acted as nucleation sites for isotropic Li growth, the MOF pores separated block-shaped Li deposits into regions for 3D matrix Li storage. This resulted in excellent Coulombic efficiency with low overpotential and dendrite-free growth during Li plating/stripping [47]. A host integrating Co-Fe binary metal selenide quantum dots into a 3D inverse opal nitrogen-doped carbon framework (3DIO FCSe-QDs@NC) was designed for application to sulfur cathodes and metal lithium anodes. The highly dispersed FCSe-QDs had good adsorption and catalytic properties, which effectively immobilized soluble LiPSs, improved diffusion conversion, and alleviated the shuttle behavior of polysulfides. At the same time, the abundant lithophilic sites of the three-dimensional ordered porous network could achieve uniform lithium deposition and uniform lithium-ion melting, thereby inhibiting dendrite growth. Taking advantage of these features, the lithium-ion battery assembled with 3DIO FCSe-QDs@NC as the main body exhibited excellent rate performance and stable cycling ability (with a decay rate as low as 0.014% over 2000 cycles under the 2 C condition). It is worth noting that under ultra-low conditions with a sulfur loading of 8.50 mg cm^−2^ and an electrolyte/sulfur ratio of 4.1 μL mg^−1^, the areal capacity of the electrode reached 8.41 mAh cm^−2^ [48]. Xu designed a novel yolk-shell nitrogen doped carbon frameworks embedded with heterostructures ZnSe-CoSe_2_ (ZnSe-CoSe_2_@NC) for both anode and cathode protection in Li-S batteries, among which the in situ formed Li_2_Se phase contributed to the Li^+^ transfer. Co and Zn guided the uniform growth of Li within the 3D framework, effectively suppressing Li dendrite growth. This design provided outstanding conductivity and stability, with a long cycle life over 1000 cycles at 2 C and a high areal capacity of 4.16 mAh cm^−2^ after 100 cycles at 0.2 C with high sulfur loading (6.08 mg cm^−2^) and lean electrolyte (4.1 μL mg^−1^) [49]. The 3D main structure has become a promising strategy to address the critical issues of the metal lithium anode, including severe volume changes and dendrite growth during battery cycling. Zhang constructed a 3D CNT framework with MnO_x_ coating using a simple hydrothermal method. Using it as the main body for the injection of molten Li, the thickness of the ultra-thin Li metal anode could be controlled to form a self-supported 10 μm ultra-thin Li metal anode, increasing the utilization of Li and limiting volume expansion. The abundant MnO_x_ nanoparticles acted as Li affinity sites, reducing the Li nucleation barrier and optimizing the electrochemical kinetics of the anode/electrolyte interface. It showed excellent lifetime extended to 2000 cycles in the symmetric cell, as well as better capacity and rate performance than the naked Li anode in the full cell [50]. Shin demonstrated through electrochemical simulations that carbon activity (lithophilic) and intergranular porosity could modulate lithium plating behavior by controlling the competitive kinetics of charge transfer and Li^+^ transport. It was shown that the lithophilicity at the bottom of the electrode was enhanced, the porosity between the particles at the top increased, and Li preferentially nucleated and then grew from the bottom upwards. The high-capacity, long-cycle main structure heterostructure prepared by two-step electrophoretic deposition spatially limit the volume change of a large number of lithium metal (6 mAh cm^−2^) with a cycle life of more than 900 cycles, which proved that the controllable heterogeneity of interfacial activity and porous structure could limit the storage behavior of lithium metal in the MOF-derived carbon host structure [51].

Wang developed a compact TiO_2_@VN (vanadium nitride) heterojunction structure with high true density (5.01 g cm^−3^), made by clever selective nitro enation, as a dual host for sulfur and Li without carbon. TiO_2_@VN exhibied special Li affinity and could serve as a Li matrix, uniformly adjusting Li nucleation and inhibiting dendrite growth, resulting in a high electrode-level volume/weight energy density for the assembled full cell [52]. Wang also designed an advanced nitrogen-doped carbon microreactor embedded with a rich Co_3_O_4_/ZnO heterojunction (CZO/HNC) as the host for the collaborative optimization of the S cathode and Li metal anode. The optimized band structure of CZO/HNC effectively promoted ion diffusion, which, in turn, promoted the bidirectional conversion of LiPSs. The lithophilic nitrogen dopant and the Co_3_O_4_/ZnO site jointly regulated the deposition of dendritic lithium. The above synergistic effect resulted in the S@CZO/HNC cathode exhibiting excellent cycling stability at 2 C, with a capacity decay of only 0.039% per cycle over 1400 cycles, while the symmetrical Li@CZO/HNC cell achieved stable lithium plating/stripping behavior over 400 h. The Li-S full cell exhibited an astonishing cycle life of over 1000 cycles [53]. Zhu designed a porous flexible self-supporting membrane composed of single-walled carbon nanotubes (SWCNTs), and the intertwined conductive network of single-walled carbon nanotubes could effectively reduce the huge volume expansion and local current density during cycling. The main body of the lithium metal anode was designed as a highly lithium-loving heterostructure (Mn_3_O_4_/ZnO@SWCNT), and the constructed p-n-type heterojunction generated a built-in electric field to promote electron transfer and Li^+^ migration. As a pre-implanted nucleation site, the strong binding of lithium-philic Mn_3_O_4_/ZnO particles to lithium atoms significantly reduce the lithium nucleation barrier. As a result of the above-mentioned synergistic effects, symmetrical cells composed of Mn_3_O_4_/ZnO@SWCNT-Li can stably remained low for more than 2500 h at 1 mA cm^−2^ and 1 mAh cm^−2^ [54].

**Table 1:** The progress and electrochemical performance of LMBs in Section 2.2.

Modification	Current Density (mA cm^−2^)	Cycle Life (h)	Number of Cycles	Sulfur Loading (mg cm^−2^)	Lean Electrolyte (μL mg^−1^)	Ref.
3D Ti_3_C_2_T_x_@Cu current collector	1	950				[42]
3D NMV	10		830			[43]
Li@/Ag||LiCoO_2_	10	90	150			[45]
Li/ZIF-8@RGO		600	350			[46]
3DIO FCSe-QDs@NC			2000	8.5	4.1	[48]
ZnSe-CoSe_2_@NC			1000	6.08	4.1	[49]
3D CNT with MnO_x_ coating			2000			[50]
MOF-derived carbon heterostructure (EPD)			900			[51]
Li@CZO/HNC		400	1400			[53]
Mn_3_O_4_/ZnO @SWCNT-Li	1	2500				[54]

## 3. Electrolyte Modification

### 3.1. Liquid Electrolyte

Liquid electrolytes have been a focal point in lithium-ion battery research for many years. The nature of the solid electrolyte interphase layer is primarily influenced by the properties of the organic electrolyte, making the optimization of salt-solvent combinations an effective strategy for generating uniform and robust SEI layers. In recent years, some novel electrolyte materials and additive have emerged [55]. Currently, mainstream modified electrolytes are mainly divided into two categories: the in situ construction of lithium-rich SEI by adding an appropriate amount of fluorinated compounds as electrolyte additives to commercial electrolytes, and the addition of sacrificial additives.

**Figure 5:**
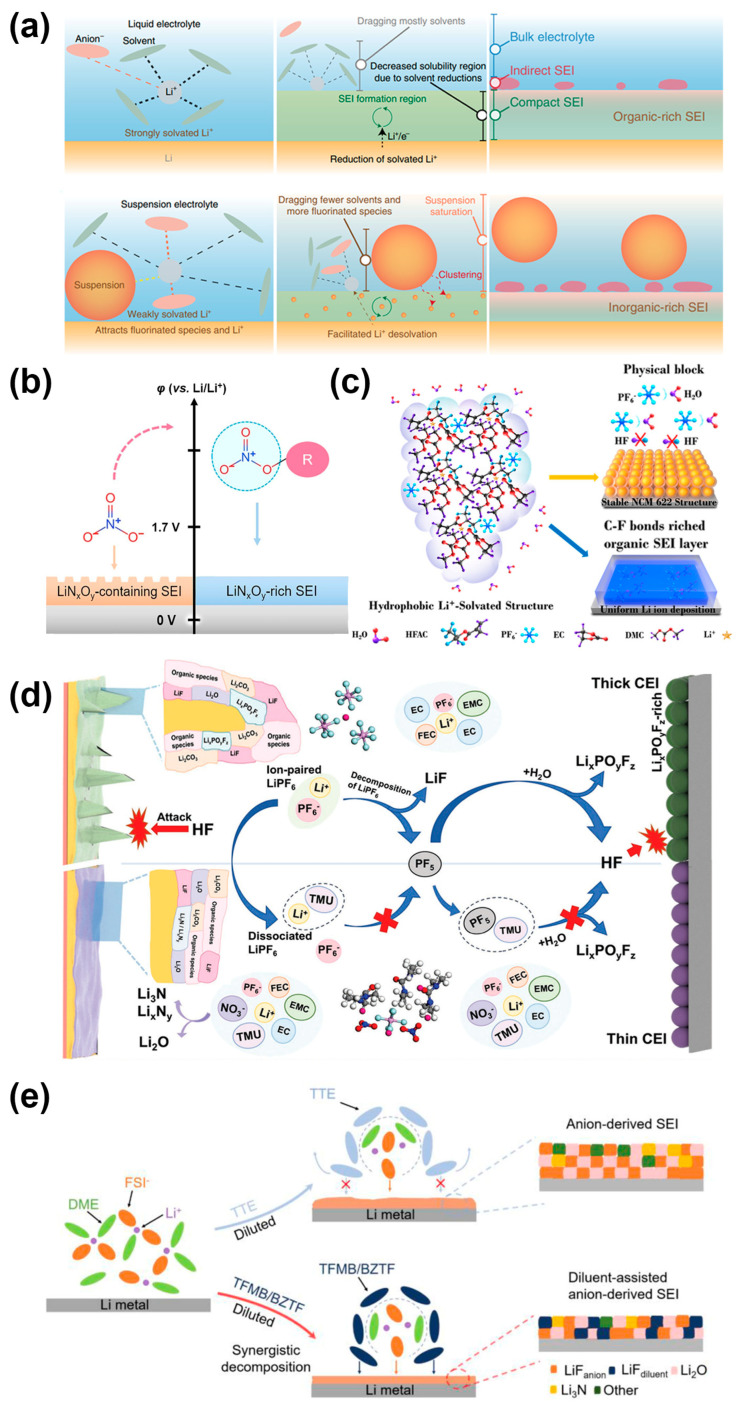
Schematic illustration of (**a**) comparison of the effects of a conventional liquid electrolyte and a suspension electrolyte on the evolution process on the Li anode [56]. (**b**) Strategy of NO_3_^−^ stabilization of SEI: reduction of NO_3_ with resonance structure to form SEI containing LiN_x_O_y_. Then, NO_3_ was modified to improve the reducibility and form LiN_x_O_y_-rich SEI [57]. (**c**) Mechanism of HFAC on the formation of organic-rich SEI layer and inhibition of LiPF_6_ decomposition [61]. (**d**) Interaction of SEI composition, solvation structure, morphology of lithium deposit, HF attack, CEI thickness, and electrochemical performance in CCE and CCE-TMU/LiNO_3_ electrolytes [62]. (**e**) The electrolyte structure and the correspondingly formed SEI in TTE-diluted and TFMB/BZTF-diluted HCEs [63].

Based on the influence of solvent polarity and donor number on Li^+^ solvation, solvent design with functional structure can increase the number of Li^+^-anion coordination and thus form inorganic species-rich SEI on the Li anode, as shown in Figure 5. Kim proposed a design strategy for suspended electrolytes to achieve the above objectives: a mixture of inorganic nanoparticles (Li_2_O) and liquid electrolytes was used as the electrolytes. The electrolytes were suspended carbonate electrolyte (SCE), suspended fluoride electrolyte (SFE), and suspended LHCE (SLHCE) and were compared. The interfacial interaction between the Li_2_O surface and the surrounding Li^+^ solvated shell achieved the interaction between Li_2_O and the surrounding liquid electrolyte. This decreased the Li^+^-solvent coordination and increased the Li^+^-anion coordination, thereby changing the Li^+^ solvation environment, forming a weak solvation environment, promoting lithium-ion mobility, and stabilizing the formation of SEI [56].

In recent years, there have been innovative modification ideas for traditional additives such as LiNO_3_. Hou amplified the positive effects of -NO_3_ by modifying it to enhance its reducibility. -NO_3_ was linked in isosorbide mononitrate to ether groups to break its resonant structure in nitrate esters, thereby significantly improving its reducibility. The decomposition of -NO_3_ resulted in SEI enriched with abundant LiN_x_O_y_ and induced even deposition of Li, which have potential commercial application prospects [57]. Zhao confirmed that LiNO_3_ could serve as an effective salt additive for carbonate electrolyte solvents and ether electrolytes. Previous efforts on high solubility of LiNO_3_ could not be achieved in carbonate ester solvents. This may have been caused by the use of mixtures of cyclic/linear carbonate ester solvents (which contained other additives) as battery electrolytes. Cyclic carbonate ester solvents (such as ethylene carbonate) could dissolve up to 0.7 M LiNO_3_ without any additives, significantly improving the reversibility of the anode and demonstrating that LiNO_3_-rich carbonate ester electrolytes could significantly enhance the stability of LMBs [58]. 

Pal developed an ether-assisted ionic liquid electrolyte to provide superior lithium metal deposition, high voltage (5 V) stability, and ultra-safety: ether solvent 1,2,2 dimethoxyethane (DME) was added to the electrolyte with a high lithium salt concentration of N-methylNpropylpyrrolidine bis(fluorosulfonyl)imide (C3mpyrFSI). This provided the better possible improvements in ionic conductivity, lithium diffusivity, and lithium plating/stripping kinetics, resulting in stable, high-speed cycling for lithium-metal/LFP (3.5 V) batteries. In the batteries with LiNi_0.8_Mn_0.1_Co_0.1_O_2_ (4.4 V) and LiNi_0.6_Mn_0.2_Co_0.2_O_2_ (4.3 V) as the cathode and Li as the anode, high Coulombic efficiency was achieved at both room temperature and high temperature. The ether ionic liquid chemistry enabled the ideal lithium plating morphology with high pile-up density, leading to minimal formation of “dead” or non-active lithium and dendrite-free long-term cycling [59].

In the non-flammable triethyl phosphate (TEP)-based electrolyte with tris(hexafluoroisopropyl) phosphate (THFP) as an additive, the polarity of the C-F bond and the abundant CF_3_ group in THFP reduced the highest occupancy molecular orbital (LUMO) and lowest unoccupied molecular orbital (HOMO) energy levels in THFP. The reduction of THFP contributed to the formation of a stable and lithium-rich SEI layer, which improved the binding capacity of PF_6_^−^ anions, significantly inhibited the growth of lithium dendrites, and reduced the decomposition of electrolytes. At the same time, THFP is also involved in the formation of a thin, CF-containing electrolyte interface layer (CEI), allowing it to provide stable cycling of the cathode at high pressures. Li||Li and THFP-added full-Li/NCM622 cells exhibited low polarization and long cycle life [60]. Another approach is to slow down Li dendrite growth by protecting the electrolyte from trace amounts of H_2_O. Hexafluoropropylene oxide (HFPO) was proposed as an additive to the electrolyte to induce a hydrophobic Li^+^-solvent coordination structure. The hydrophobicity of HFPO, as well as the olefin groups and non-polar perfluorocarbon chains (-CF_2_CF_2_CF_3_), protected symmetrical cells from a slight attack by H_2_O and avoided the formation of ionic insulation decomposition products on the lithium anode and cathode. The LUMO and HOMO corresponding to HFPO made it reduce/oxidize on both the lithium anode and cathode, forming a rich organic SEI film and cathode electrolyte interface layer (CEI), which could adapt to the evolution of the anode/cathode structure [61]. Metal ions and organic polymers were used as electrolyte additives to effectively control lithium-ion deposition and alleviate lithium dendrite problems, thereby improving the coulombic efficiency and stability of lithium-ion batteries. When lithium nitrate additives and small amounts of tetramethylurea (TMU) were introduced into commercial carbonate electrolytes as multifunctional co-solvents, normally insoluble NO^3−^ ions can be incorporated into the solvation structure of Li^+^ ions to form conductive and stable SEIs. The solvation structure was controlled, and the scavenging effect could be used to inhibit the production of HF [62]. Fluorinated aromatic diluent was introduced into the high-concentration electrolyte, where the diluent was paired with anions to facilitate the formation of a uniform and robust SEI. Their synergistic effect on the SEI endowed Li metal with extraordinarily high coulombic efficiency as high as 99.8% [63].

### 3.2. Solid-State Electrolyte

SSBs can increase energy density and eliminate the safety risks associated with flammable liquid electrolytes found in traditional lithium-ion batteries. Companies in the automotive and battery industries are already planning research and development in the field of SSBs [64,65]. Yi coated a non-flammable gel layer on the surface of CPE and designed a multilayered hybrid electrolyte (MHE) to eliminate the safety hazards of solid-state lithium-ion batteries. The hydrogen bonding interaction between F and H atoms allowed the solvent to easily anchor the TFSI^−^ anion in the liquid electrolyte. The gel layer was therefore well compatible with the lithium metal anode. Coupled with the good mechanical properties of CPE, the prepared MHE had a wide electrochemical window, a high ion mobility number, and ionic conductivity [66]. Zhang discovered that the blending of different polymers significantly enhanced the high-temperature deformation resistance of materials, and the use of dual-salt could strengthen the migration of lithium in solid substrate. As a result, a solid polymer electrolyte composed of a blend of polymers with polyethylene oxide (PEO) and polyvinylidene difluoride (PVDF) was developed, which is suitable for large-scale production and an ideal choice for high-temperature solid polymer batteries. In the PEO/PVDF blend matrix, the battery performance is excellent, and the presence of PVDF can maintain the shape of the membrane, avoid short circuits, and improve the safety of battery operation at high temperatures [67]. Ma designed a novel scalable, ultra-thin, high-temperature-resistant solid electrolyte (SPE) to provide a stable SSE/lithium-containing LiF and Li_3_N interface. The SPE included an electro spun polyacrylonitrile (PAN) matrix and a polyethylene oxide (PEO)/lithium salt-ion conductor with excellent mechanical strength, which effectively inhibited lithium dendrites, prevented short circuits, and improved the performance of symmetrical lithium-ion batteries [68].

Kang produced SE with high ionic conductivity from a high dielectric constant polymer that could strongly interact with lithium salts. This polymer had a significant dipole moment that directed the transport of lithium ions (Li^+^) along the chain, facilitating their continuous jumping in the solid polymer electrolyte (SPE) system [69]. In the solid electrolyte layer, the low porosity of oxide-based SE requires very high sintering temperatures to achieve, accompanied by poor interfacial contact. However, the most typical MOF-based SEs have high production costs, redundant preparation processes, and poor thermal stability, which is the most critical issue. Additionally, the relatively low ionic conductivity of both oxide-based SE and MOF-based SE at room temperature greatly limits the critical current density and high-rate performance of the battery, which is one of the great challenges in battery performance optimization. At present, most of the strategies using liquid alkali metal anode/interface layer have achieved some success in inhibiting alkali metal dendrite growth and improving the cycling stability of the battery. The highly conductive liquid anode has a strong ability to dissolve alkali metals even after nucleation is completed, and it is an effective strategy to fundamentally inhibit dendrite growth. However, attention needs to be paid to the safety performance of SEs and liquid alkali metal anodes/interface layers to prevent short circuits caused by liquid flow. Considering the above, Peng proposed a new system that combines a sulfide-based solid electrolyte with a liquid metal lithium anode (Li-Bp-DME), which further improved battery performance while retaining the inherent advantage of Li-Bp-DME in suppressing lithium dendrite nucleation and growth. Compared with other SEs, these sulfide-based SEs had superior ionic conductivity at room temperature and better high-rate charge-discharge performance, which could almost completely block the penetration of Li-Bp-DME, and greatly improved the cycling stability and lifetime. The addition of a polymer interface layer in the system further improved the compatibility and stability of Li-Bp-DME with sulfide-based solid electrolytes at the interface. The new battery structure using sulfide-based SEs, liquid metal lithium anodes (Li-BpDME), and interface optimization achieved long cycle life and record-breaking high critical current density up to 17.78 mA cm^−2^ [70]. Li_7−x_PS_6−x_Cl_x_ (x = 0.6, 1.0, 1.3, 1.45, and 1.6) electrolytes were characterized physically in situ and in situ, chemical, and electrochemical studies. During the synthesis process of the SE, the distribution of Cl and the cooling process have a significant influence on the microstructure, interface composition, and morphology evolution of the Li|SE interface. For SEs with an appropriate amount of Cl, Cl atoms form interconnected LiCl nanoparticles on the surface of the SE grains, which can extend into a LiCl framework. This is beneficial for Cl ions to migrate to the interface during electrochemical cycling, thereby improving the cycling performance of the battery [71].

## 4. Interface Design Modification

### 4.1. Separators

**Figure 6:**
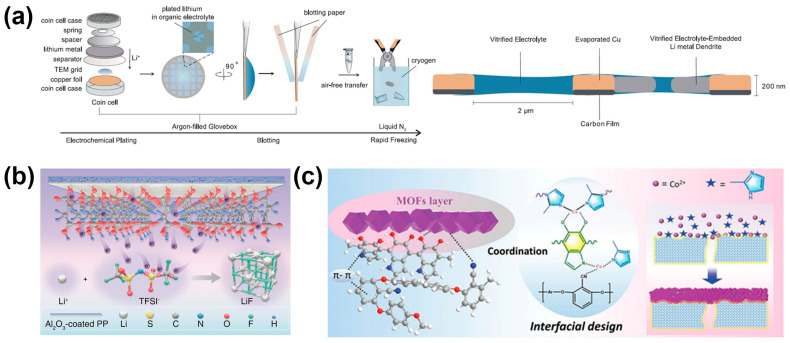
Separators: Schematic of (**a**) preparation process and schematic cross-section of vitrified specimens [73]. (**b**) Synthesis of LiF-rich SEIs: SAMs with carboxy-terminal groups accelerate the reduction process of LiTFSI by dipole moment directed electron provision [74]. (**c**) Internal construction of sandwich-structured MOFs/NA/MOFs hybrid separators [75].

**Figure 7:**
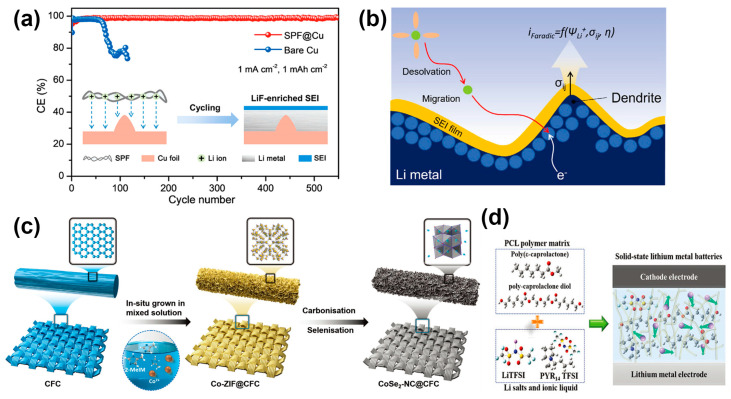
Artificial SEI: schematic diagram of (**a**) the SPF-mediated Li metal anode before and after cycling [79]. (**b**) Electrodeposition of Li at the interface between the bulk metal electrode and SEI (electrochemical kinetics and mechanical stresses) [80]. (**c**) Preparation route for CoSe_2_-NC@CFC [82]. (**d**) The PIA-SPE [83].

Regarding the growth of anode metal dendrites, functional separators and interlayers are also an innovative method for defect remediation. Common methods include introducing a protective layer as an artificial SEI (anode) and comprehensive improvement of the wetting of the electrolyte and the uniformization of ion flux (anode and cathode) [72]. Separators are widely studied as a better alternative that involves the preparation of a special functional membrane and the use of its interaction with free solvent molecules to filter out the free molecules and obtain a high concentration of electrolyte after dissolution. The absence of expensive and toxic additives to the electrolyte is the biggest advantage of the separator. Lithium metal batteries assembled with functional separators can generally operate efficiently at room temperature, improving battery safety, as shown in Figure 6 and Figure 7. Figure 6 summarizes the schematic diagram of Separators, and Figure 7 summarizes the schematic diagram of Artificial SEI.

Zhang used thin-film vitrification to retain the cell’s electrode-electrolyte interface in its native organic liquid electrolyte environment. Using cryogenic scanning transmission electron microscopy characterization, the complete structure and chemical properties of the metal lithium battery interface were studied, and it was found that SEI expansion could be effectively alleviated by increasing the electrode/electrolyte interface area and changing the composition and thickness of SEI. Since the presence of solvents in SEI reduces the distance required for electron tunneling during electrolyte decomposition, solvent diffusion may play a more important role in the ongoing formation of SEI than other factors [73]. Liu designed self-assembled monolayers (SAMs) with high-density and long-range ordered polar carboxyl groups connected to alumina-coated separators. They could provide a strong dipole moment while providing excess electrons to accelerate the degradation kinetics of carbon-fluorine bonds in Li bis (oxalato) borate. The resulting SEI rich in LiF nanocrystals promoted rapid transfer of Li^+^ and inhibited dendrite growth. In particular, the SAMs significantly enhanced the cycling performance of the whole cell under high-cathode loading, limited lithium excess, and poor electrolyte conditions [74]. A sandwich MOF/PEN@PDA/MOF multifunctional membrane was fabricated through the in situ growth of ordered anionic MOF layers on both sides of a pre-modified polyether nitrile (PEN) porous super engineering film by polydopamine pre-treatment. The optimized membrane completed the task of guiding the migration of lithium ions, limiting the free migration of anions, balancing the internal electric field, and prolonging the nucleation time of lithium dendrites, resulting in a high Li-ion transfer number of 0.81. Moreover, the multifunctional membrane showed better thermal stability than traditional polypropylene membranes. With reduced interfacial side reactions, the optimized battery exhibited highly stable cycles over 500 h. The assembled LFP/Li battery with the optimized membrane even showed steady cycling performance and a coulombic efficiency as high as 98% even at 90 °C [75]. The unique characteristics of covalent organic frameworks (COFs) make it a potential separator to address the issues mentioned above. COFs have a strong affinity with Li+, but the relationship with solvent molecules in electrolyte is exclusive. According to the above principle, Yang synthesized a self-loading COF membrane (TPB-BD(OH)_2_-COF) as a separator for lithium metal batteries, which realized the aggregation of electrolyte and reduced the side effects of free solvent and lithium metal. The separator worked well even under extreme conditions (60 °C) [76]. Wang used MoP@NC composites modified with N-doped thin carbon layer (MoP@NC) and porous carbon nanofibers (PCNF) to coat the surface of the Celgard separator in an attempt to solve the problem of low sulfur utilization rate and short cycle life of lithium-sulfur batteries. The MoP@NC materials extracted from molybdenum-based MOFs (Mo-MOFs) had abundant pore volumes and multiple catalytic sites, and they presented rod-like morphologies with uniform structure and stable properties. PCNFs and N-doped thin carbon layers could effectively alleviate volume expansion, buffer electrolytes, and capture LiPS. When used as a modified layer on a separator, MoP@NC/PCNFs-based cells had optimized lithium and sulfur affinities, achieving ideal sulfur electrochemistry, and could be applied to high-performance Li-S batteries [77].

### 4.2. Artificial SEI

The Li deposition process is primarily controlled by the SEI on the metal surface, making the design of artificial SEI a crucial approach for regulating lithium electrodeposition. The main objective of designing an SEI layer on the metal lithium anode is to enhance the ionic conductivity of the SEI, thereby reducing the loss of lithium ions from the metal surface. Additionally, the construction of artificial SEI with improved mechanical properties can also suppress mechanical failures (fracture/cracking) caused by dendrite growth or interfacial displacement [78]. 

Ju developed a versatile artificial SEI that uses commercially produced soy protein fibers (SPF) to stabilize the metal lithium anode. The abundance of polar functional groups in its protein molecules could promote consistent lithium-ion flux and induce uniform lithium deposition. Lithium-philic and porous SPF could significantly alleviate the ion concentration gradient between electrodes, contributing to the deposition of biomimetic lithium along the fiber structure. The introduced SPF promoted the formation of SEI structures rich in LiF nanocrystals after cycling, thereby achieving low interfacial impedance and fast charge transfer kinetics, greatly reducing the concentration gradient of lithium ions, and achieving uniform distribution of lithium ions [79]. 

It is generally accepted that dendrite growth is thought to be related to the average stress during plating/stripping and the elastoplastic deformation of lithium metal and SEI. Theories such as the Butler–Volmer reaction kinetics of local current distribution, the space charge layer, and the mass transfer limit near the electrode surface have been proposed to explain the mechanisms of electrochemical and mechanical processes. Liu developed an electrochemical-mechanical model involving electrochemical kinetics and mechanics to simulate the performance manipulation of artificial SEI to achieve uniform lithium electrodeposition, mainly developed from a modified version of the Butler–Volmer equation. The model included an electric field, a stress field, and a lithium-ion concentration field, and the author described the process of electrodeposition at the interface between SEI and lithium metal. The results showed that the ionic conductivity and Young’s modulus of SEI played an important role in the deposition behavior of lithium battery. If the ionic conductivity of SEI was increased above the critical level, the stress concentration was significantly reduced, and Li was preferentially deposited. When the SEI had sufficient mechanical strength (Young’s modulus of about 4.0 GPa), the inhomogeneous deposition was reduced. On top of this, increasing the ionic conductivity could further improve the performance of the battery [80]. Zhang used an electrochemical phase field (PF) model to simulate inhomogeneous lithium deposition in porous lithium metal anodes. In the four factors studied: the porosity of the lithium metal anode, the diffusion coefficient of lithium ions, the reaction constant of lithium deposition, and the structural electrode with porosity gradient, it can be found that the lithium deposition in the pores can be improved by increasing the diffusion coefficient of lithium ions and designing lithium metal anodes with reaction constant gradient and porosity gradient [81]. On carbon fiber cloth (CoSe_2_-NC@CFC), a layered 3D structure using CoSe_2_ nanoparticles to anchor nitrogen-doped carbon nanosheet arrays was established to regulate lithium nucleation/plating processes and stabilize the electrolyte anode interface. Due to the enhanced lithium affinity of CoSe_2_-NC, Li_2_Se and Co nanoparticles formed in situ during the initial nucleation process, as well as the large pore space, could induce uniform Li nucleation/plating, optimize the SEI, and reduce volume changes [82]. 

Zhang selected 1-butyl-1-methylpyrrolidine bis (trifluoromethanesulfonyl) imide (BMI-TFSI) as the initiator to promote the formation of an artificial SEI protective layer between PIA-SPE and the metal lithium anode, providing it with more TFSI-anionic groups. A stable SEI layer (consisting of LiF, Li_2_S_x_, and Li_3_N) was formed in situ on the electrolyte/Li surface, which promoted uniform deposition of lithium [83]. Li achieved an ideal lithium metal anode by the reaction of porous polymer backbone macromolecular brush (polyethylene glycol) methyl methacrylate, super-cross-linked polystyrene nanospheres (representing xPCMS-g-PEGMA) and single-ion conductive lithiation Nafion. The porous xPCMS core with a rigid hyper-crosslinked framework significantly improved the mechanical strength, providing sufficient channels for rapid ion conduction. Flexible PEGMA and lithiated Nafion polymers formed a structurally stable artificial protective layer that provided uniform Li diffusion and high Li migration number. The structure enabled ultra-high current density, unprecedented reversible lithium plating/stripping, and ultra-long-term stable cycling [84].

### 4.3. Buffer Layer

Aiming at the problems of poor physical contact and serious interface reaction at the negative electrode interface, the construction of a buffer layer on the surface of the solid electrolyte can improve the contact between the negative electrode interface and alleviate the interface chemical reaction [85]. Shen developed reduced Graphite oxide (RGO) coupled porous MoO_2_-Mo_3_N_2_ heterojunction nanoribbons decorated copper collector (GMM@Cu) used for high-performance LMBs. The composite collector with functional interface had rich nucleation sites. Electroplating Li_2_O and Li_3_N, which are lipophilic to lithium, effectively accelerated the Self-diffusion of lithium ions in BIEF and promoted the uniform electrodeposition of lithium. The synergistic effect of the built-in electric field formed by good interface contact and the dense, lithium-rich SEI layer promoted charge transfer and ion diffusion and improved the uneven lithium-ion flux to suppress the growth of dendrites. In addition, the introduction of flexible Graphene layer improved the structural integrity and electron transport dynamics [86].

Zhang constructed a value-gradient iron-based protective layer on the Li anode and applied it to various batteries, which proved the superiority of the protective layer in the protection of lithium anode. The protective layer consisted of an outer layer containing Fe^3+^/Fe^2+^ and an inner layer containing Fe^0^, which not only isolated the lower layer of lithium metal from the corrosive carbonate electrolyte, but also stored lithium evenly during the plating process to inhibit lithium dendrite growth, exhibiting dendrite-free lithium plating/stripping behavior. The Li symmetrical battery ran stably for 1000 h at 1 mA cm^−2^ and 1 mAh cm^−2^, and it even survived for 380 h at 30 mAh cm^−2^ ultra-high capacity. The lithium anode achieved high performance in LiFePO_4_ batteries with 1600 and 1000 cycles of stable operation at 5 °C, respectively. Its high-load LiCoO_2_ cells also exhibited excellent cycling stability and rate capability [87].

## 5. Conclusions and Prospects

Lithium anode protection is a critical aspect of ensuring the stability and safety of lithium-ion batteries. Advances in SEI engineering, composite solid electrolytes, and electrode architectures have shown great potential in overcoming the challenges associated with lithium metal anodes. Continued research efforts to address the remaining challenges will pave the way for the widespread application of high-energy-density lithium-metal-based batteries in various industries. Advanced Li metal anode, electrolyte modification, and interface design modification collaboratively operate to solve the technical problems faced by the Li metal anode. These strategies not only solve the single problem but can usually solve several problems at the same time. Even so, there are still some challenges to improving the performance of lithium anodes in lithium batteries. First of all, the performance of the protective material itself needs to be further improved, and it is necessary to develop the composite properties of new materials or materials, exploring new combinations and new functions, such as the combination of special films and bonds. Secondly, although a modification strategy with high comprehensive performance has been developed, the problem of dendrite growth has not been completely solved, and its nucleation and growth mechanism lack sufficient understanding, so further theoretical research is needed using machine learning, artificial intelligence, and theoretical calculation. For example, electrochemical mechanical modeling, multi-field simulation, electrochemical phase field, and other means are used to analyze and predict the inhomogeneous lithium deposition process and control the factors, so as to guide the actual deposition behavior and create better performance. Finally, due to the fact that most of the dendrite growth processes are not observed in time, there is a lack of research on the in situ evolution mechanism of the lithium metal anode interface, and more appropriate in situ analysis techniques need to be developed to analyze and understand the evolution behavior of the interface. Under the development trend of button cell battery to pouch battery, how to better contact with other components in the all-solid-state battery without affecting the interaction is a problem that lithium metal anode still needs to face. Researchers are needed to create more novel SEIs or electrolytes. Regarding the performance improvement strategy of lithium anode in lithium batteries, it can be developed in theoretical calculation and comprehensive regulation in the future.
